# 3D similarities between the binding sites of monoaminergic target proteins

**DOI:** 10.1371/journal.pone.0200637

**Published:** 2018-07-20

**Authors:** Gabriel Núñez-Vivanco, Angélica Fierro, Pablo Moya, Patricio Iturriaga-Vásquez, Miguel Reyes-Parada

**Affiliations:** 1 Centro de Bioinformática y Simulación Molecular, Universidad de Talca, Talca, Chile; 2 Escuela de Ingeniería Civil en Bioinformática, Universidad de Talca, Talca, Chile; 3 Pontificia Universidad Católica de Chile, Santiago, Chile; 4 Instituto de Fisiología, Universidad de Valparaíso, Valparaíso, Chile; 5 Centro Interdisciplinario de Neurociencia de Valparaíso CINV, Universidad de Valparaíso, Valparaíso, Chile; 6 Facultad de Ingeniería y Ciencias, Universidad de la Frontera, Temuco, Chile; 7 School of Medicine, Faculty of Medical Sciences, University of Santiago de Chile, Santiago, Chile; 8 Facultad de Ciencias de la Salud, Universidad Autónoma de Chile, Talca, Chile; Universita degli Studi di Roma Tor Vergata, ITALY

## Abstract

The study of binding site similarities can be relevant to understand the interaction of different drugs at several molecular targets. The increasing availability of protein crystal structures and the development of novel algorithms designed to evaluate three-dimensional similarities, represent a great opportunity to explore the existence of electronic and shape features shared by clinically relevant proteins, which could assist drug design and discovery. Proteins involved in the recognition of monoaminergic neurotransmitters, such as monoamine transporters or monoamine oxidases (MAO) have been related to several psychiatric and neurological disorders such as depression or Parkinson’s disease. In this work, we evaluated the possible existence of similarities among the binding sites of the serotonin transporter (SERT), the dopamine transporter (DAT), MAO-A and MAO-B. This study was carried out using molecular simulation methodologies linked to the statistical algorithm PocketMatch, which was modified in order to obtain similarities profiles. Our results show that DAT and SERT exhibit a high degree of 3-D similarities all along the pathway that is presumably involved in the substrate transport process. Distinct differences, on the other hand, were found both at the extracellular and the intracellular ends of the transporters, which might be involved in the selective initial recognition of the corresponding substrate. Similarities were also found between the active (catalytic) site of MAO-A and the extracellular vestibule of SERT (the S2 binding site). These results suggest some degree of structural convergence for these proteins, which have different functions, tissue distribution and genetic origin, but which share the same endogenous ligand (serotonin). Beyond the functional implications, these findings are valuable for the design of both selective and non-selective ligands.

## Introduction

Monoaminergic neurotransmitters exert their actions by interacting with diverse protein targets in their synapses. Thus, serotonin (5-HT), norepinephrine (NE) and dopamine (DA), activate either metabotropic or ionotropic receptors (e.g. β-adrenoceptors or 5-HT_3_ receptors, respectively), are catabolized by monoamine oxidase and/or catechol-*ο*-methyltransferase (in the case of NE and DA) and are pumped back into their synaptic terminals by selective transporter proteins. Furthermore, these neurotransmitters are accumulated into synaptic vesicles inside the nerve terminals by specific transporters, and even within noradrenergic neurons the presence of the enzyme DA-β-hydroxylase can be considered as an additional site for the interaction of DA [1,2]. Moreover, several other compounds, including therapeutically useful and widely used recreational drugs which bind to more than one of these receptor proteins have been described [2,3].

Even though this promiscuous interaction does not usually grab attention, it should be kept in mind that monoamine receptors, metabolic enzymes and transporters belong to different protein families, with highly diverse functionality, genetic origin and structure. That is the case, for instance, of the 5-HT and DA transporters (SERT and DAT, respectively) and monoamine oxidases. Although both types of proteins are involved in terminating the actions of 5-HT or DA at their synapses, their cellular localization, mechanism, structure and function are markedly different. Thus, human DAT and SERT are plasma membrane proteins which belong to the neurotransmitter/sodium symporter (NSS) family [4,5]. The active reuptake of the corresponding monoamine into the neurons (or glial cells) against a concentration gradient is carried out by coupling the flow of neurotransmitters to that of sodium and chloride (and also to the counter-transport of potassium in the case of SERT) [6]. Much of the structural knowledge about human DAT and SERT transporters is based on the crystal structures of the LeuT (and homology models derived from them), a homologue Na^+^-coupled transporter from the bacterium *Aquifex aeolicus* [7–9]. Furthermore, the crystal structures of the DAT from *Drosophila melanogaster*, and the human SERT have been recently reported [10,11]. In general terms, these structures revealed that these transporters contain a shot glass-shaped bundle of 12 transmembrane helices, which are connected by six extracellular and five intracellular loops. A central binding site is located approximately halfway across the membrane bilayer. At this site, both substrates and inhibitors [10–12], establish an ionic interaction between the ligand amino group and the carboxylate of a conserved aspartate (D46 in DAT from *Drosophila*, D98 and D79 in human SERT, and DAT, respectively). In these transporters, the majority of residues at this binding site are hydrophobic, although a few polar residues are able to form strong interactions with the substrates. All the crystal structures (as well as homology models, [13]) show the existence of a secondary binding pocket termed the extracellular vestibule, since it is located closer to the extracellular side of the transporter. Although in LeuT this site can accommodate selective 5-HT reuptake inhibitors or tricyclic antidepressants [14–16], more recent data using engineered LeuT or actual MA transporters, have unequivocally shown that these drugs inhibit DAT or SERT by acting at the central binding site [17,18]. Nevertheless, pharmacological, mutagenesis and structural studies [17,19,20] have shown that the extracellular vestibule is an allosteric site, which can positively or negatively modulate activity at the central binding site.

On the other hand, monoamine oxidase (MAO), which in humans exists in two isoforms termed MAO-A and MAO-B, are outer mitochondrial membrane-bound flavoproteins, with the FAD cofactor covalently bound to the enzyme. 5-HT and 2-phenylethylamine are selectively oxidized by MAO-A and MAO-B respectively, while DA is a non-selective substrate of both enzyme isoforms [21,22]. MAOs oxidize monoamines through a reaction that involves an α-C-H bond cleavage of the substrate and the concomitant reduction of the flavin cofactor, which is reoxidized by molecular oxygen producing hydrogen peroxide [21,22]. Since 2002 a series of articles showing high-resolution structures of human or rat MAOs have been published [23–27], allowing a detailed comparison of the overall structures of both isoforms and their active sites [28]. Thus, the substrate/inhibitor binding site of both isozymes can be described as a pocket lined by the isoalloxazine ring and several aliphatic and aromatic residues. A critical role of Y444, Y407, G215 and I180 of MAO-A (Y435, Y398, G206 and L171 being the corresponding residues in MAO-B) in the orientation and stabilization of the substrate/inhibitor binding can be inferred from the X-ray diffraction data. In the case of MAO-B, the substrate/inhibitor binding site is a cavity (a flat entity of 420 Å^3^ in volume, termed the “substrate cavity”) which can be distinguished, in some cases, from another hydrophobic entity (290 Å^3^ in volume, termed the “entrance cavity”) located closer to the protein surface. In contrast, human and rat MAO-A’s differ from human MAO-B in that they have only a single cavity, although it has been suggested that a two-cavity system could also exist in MAO-A [29].

The interaction of monoamines with their targets relies primarily on the shape and electronic complementarities between the ligand(s) and the receptor(s) binding site(s). Therefore, it seems reasonable to assume that all proteins targeted by a given monoamine should have certain similarities of these features at their binding sites. The availability of the crystal structures of either some of these proteins [10,30–32] or their insect homologues [11,12,33,34] as well as the development of an increasing number of algorithms aimed to evaluate the similarities between the binding sites of related and unrelated proteins [35–38], provide an exceptional framework to test this hypothesis.

Based on these precedents, in the present work we evaluated the likely existence of similarities among the binding sites of SERT, DAT, MAO-A and MAO-B. Beyond the mechanistic or functional clustering implications, we anticipate that the study of binding site similarities (and differences) among these proteins can be insightful for the rational design of selectively and non-selectively acting compounds.

## Materials and methods

### Molecular structures

The crystallographic data of human MAO-A ([31]; 3.1 Å resolution), MAO-B ([24]; 1.7 Å resolution) and SERT ([10]; 3.1 Å resolution) were taken from the Protein Data Bank (PDB; PDBcodes: 2BXS, 1OJA and 5I6X respectively). A homology model of the human DAT was built as previously described [39,40] using the crystal structure of the DAT from *Drosophila melanogaster* ([11]; 2.9 Å resolution, PDB code 4XPA) and the human SERT as templates.

In all cases, the corresponding transmembrane segments were embedded in a hydrated palmitoyl-oleyl-phosphatidyl-choline (POPC) bilayer membrane, solvated in a water box (type TIP3), and ions were added creating an overall neutral system in approximately 0.2M NaCl. The final systems were subjected to a molecular dynamics (MD) simulation for 5 ns using NAMD 2.6 [41]. The isobaric-isothermal ensemble (temperature of 310 K and 1 atm) was used to perform MD calculations. Periodic boundary conditions were applied to the system in the three coordinate directions. The simulation time was sufficient to obtain an equilibrated system (RMSD < 2 Å; Figures A, B, C and D in S1 File). Stereochemical and energy quality of the homology model was evaluated using the PROSAII server [42] and Procheck [43] (Figures E and F in S1 File). Due to the size and complexity of the systems, in the case of SERT and DAT, MD simulations were performed applying some constraints to the backbone of the proteins.

### Dummy atoms

An aspect that should be solved before comparing the ligand binding sites is that, because of their structure and function, both transporters and MAOs probably do not have a single ligand binding site. Indeed, both in SERT/DAT and in MAO-B (and probably in MAO-A [44]), a two-cavity system covered by a flexible loop has been described as the presumable path that has to be traveled by the ligand (substrates or inhibitors) in order to reach its "final" binding site [28,45,46]. Moreover, pharmacological, mutagenesis and structural studies [19,20,47,48] have shown that the extracellular vestibule/entrance cavity (according to the nomenclature used for the secondary binding site found in transporters and MAOs, respectively), is an allosteric site which can positively or negatively modulate activity at the orthosteric binding site.

**Fig 1:**
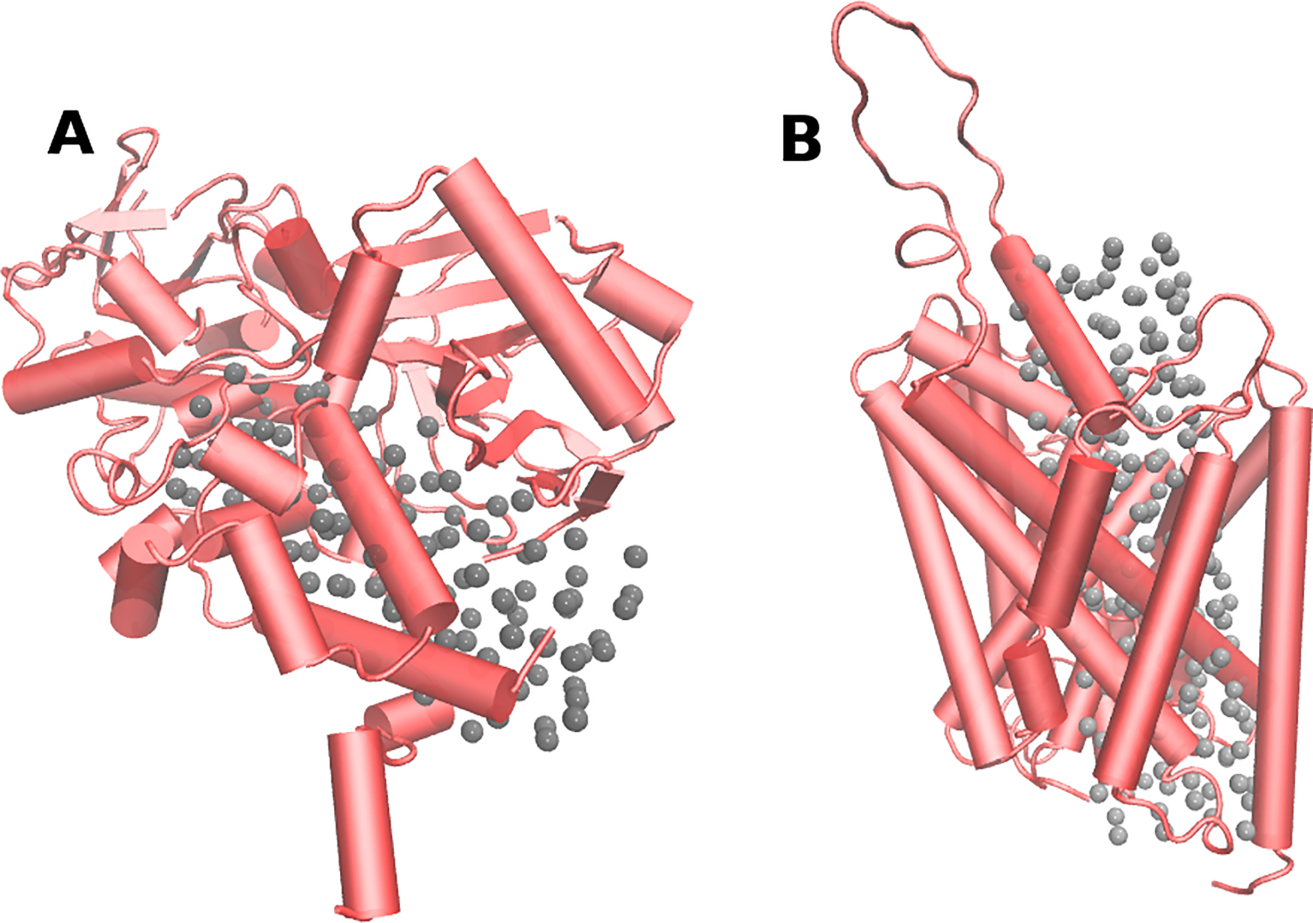
**Dummy atoms located in: (A) MAO-A and (B) SERT**. Both proteins are shown in red with a ribbon format. Each gray sphere represents a dummy atom.

Then, the pathway traveled by the ligand in these targets might contain several binding sites, and similarities between the different proteins might appear in any of them. Therefore, in order to consider all possible ligand binding sites and to explore their similarities in detail, we decided to fill SERT, DAT, MAO-A and MAO-B cavities with "dummy atoms". It should be noted that the coordinates of dummy atoms, which in size correspond to a hydrogen atom, were used only as spatial references for the similarity measurements (see below), and they affect neither the structure nor the function of the protein. For MAO structures, a rectangular box of 15 Å x 15 Å x 30 Å was defined, and its center was located in the middle of the substrate/inhibitor cavity. 118 dummy atoms were located uniformly in this space (Fig 1A). The rectangular box defined for SERT and DAT was of 15 Å x 15 Å x 60 Å and 192 dummy atoms were located in this space with a uniform distribution (Fig 1B).

### Scoring of ligand binding sites similarity using dummy atoms

**Fig 2:**
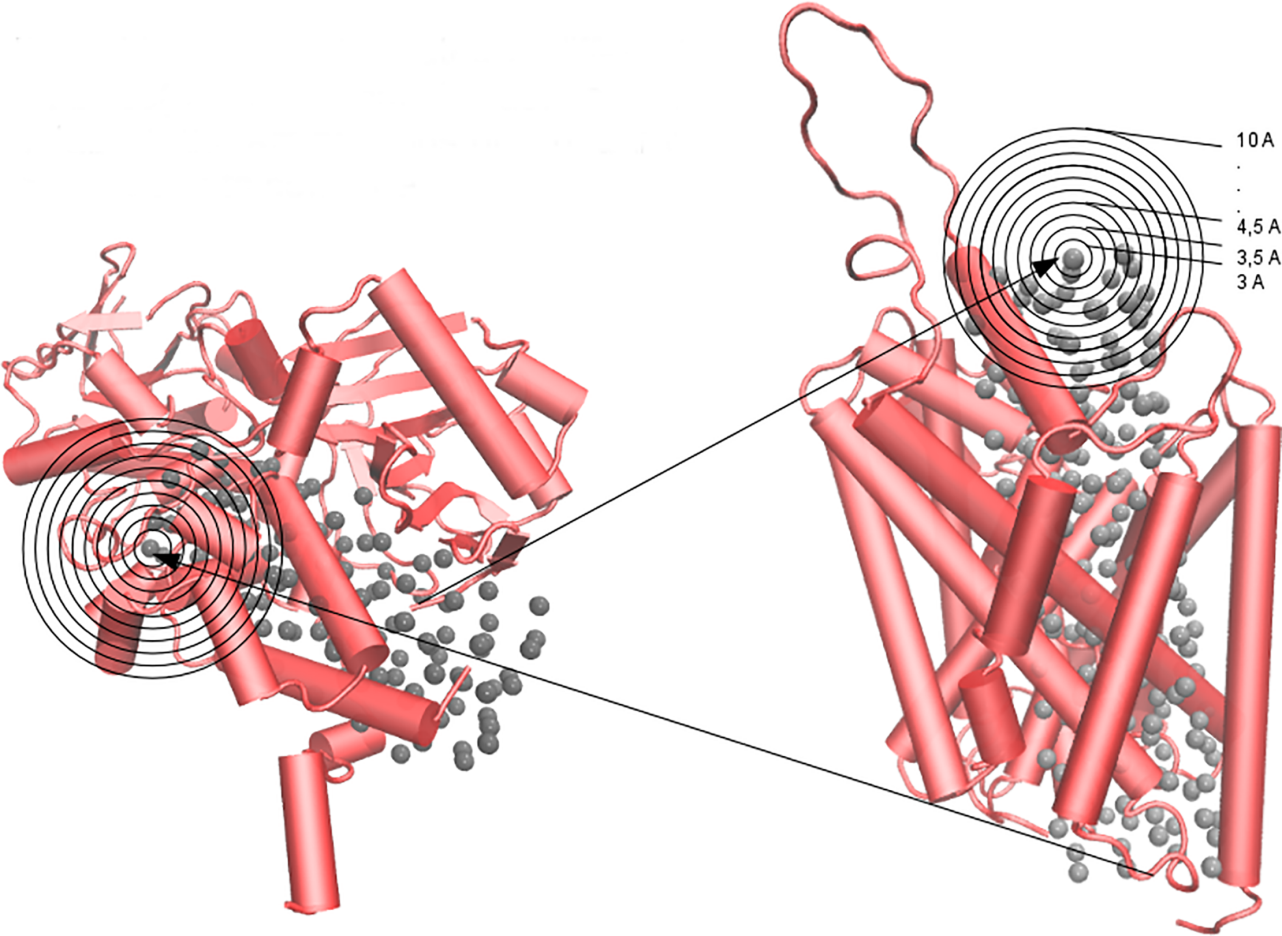
Representation of the all-against-all procedure applied to compare all possible binding sites defined by the dummy atoms (grey spheres). Each black circle denotes a different distance measured. MAO-A (left) and SERT (right) are shown in red with a cartoon format.

To evaluate the similarity between the ligand binding sites at the proteins of interest we used the Pocketmatch algorithm [49]. All aspects involved in binding site comparisons followed the procedure published in the original article describing the algorithm with minor modifications [50,51]. Briefly and as previously described [51], each binding site was considered as that determined by the residues for which one or more atoms surround a dummy atom (a crystallographic ligand in the original report) at a given distance (distances from 3 Å to 10 Å from the dummy atoms were considered). Each residue was classified into one of five groups (tag 1–5), taking into account its chemical properties. Then, each residue was represented as a set of three points corresponding to the coordinates of the α-C, the β-C, and the centroid coordinates of the side chain. Distances between every three points of each residue in the binding sites were measured. All computed distances were sorted in ascending order and stored in sets of distances organized by type of pairs of points and type of pairs of tags. The sorted and organized distances were then aligned and compared using a threshold of 0.5 Å, which was established considering the natural dynamics of biological systems. The similarity between sites, referred to as the PMScore, was evaluated by scoring the alignment of the pair of sites under comparison. Thus, the PMScore represents the percentage of the number of "matches" calculated over the maximal number of distances computed for each binding site. A PMScore of 0.5 (50%) or higher was considered as indicative of similarity between binding sites. To evaluate the similarity between the cavities of SERT and DAT where dummy atoms were inserted, each binding site surrounding the 192 dummy atoms in SERT (considering 15 distances from the atom, from 3 Å to 10 Å) were compared with the corresponding dummy atom binding sites in DAT (*i*.*e*., 192 x 192 x 15 = 552.960 measurements of PMscore were made). To compare SERT/DAT with MAO-A/MAO-B, 339.840 determinations of PMscore were made between the binding sites surrounding the 118 dummy atoms in the enzymes and those of the 192 dummy atoms in the transporters. Fig 2 illustrates how the pairs of dummy atoms were selected in MAO-A and SERT for the comparisons. Finally, the mean of the PMScores obtained after comparing each pair of dummy atom binding sites (3–10 Å) was used to determine the similarity between the binding sites in the analyzed proteins.

### Scoring of binding site similarity in MAO-A and SERT using a promiscuous ligand

In order to analyze the similarities found in a more realistic approach, we compared the characteristics of the binding sites of 4-methylthioamphetamine (MTA) in MAO-A and SERT. MTA is a non-selective ligand that exhibits affinity for both proteins in the low micromolar range [52,53]. To this end, and given that MTA is a 5-HT releasing agent and apparently a SERT substrate [52], we used steered molecular dynamics (SMD; see below) to simulate the transport of MTA along the substrate pathway of SERT. Thus, several conformations of the SERT-MTA complex were obtained, and each one was considered as defining a putative binding site of the drug at this protein. On the other hand, as MTA is a competitive MAO-A inhibitor [54], the drug was docked into the active site of the enzyme as previously described [54]. Finally, the binding sites of MTA at SERT and MAO-A were compared using the Pocketmatch algorithm, where every similarity determination between the MTA/MAO-A complex and each MTA/SERT conformation was evaluated at 70 distances (from 3 Å to 10 Å adding 0.1 Å in all iterations).

### Steered molecular dynamics simulations (SMD)

Through SMD, a time-dependent external force was applied to simulate the uptake of MTA by SERT. During the simulation, we calculated the force exerted as well as the external work performed on the system. Each simulation lasted 3 ns, which was sufficient to observe almost the entire ligand uptake process (see discussion). The mean force for each step of the simulation was calculated by averaging the outcomes of 4 independent runs. Before simulations, the MTA-SERT complex was inserted into a POPC membrane and solvated with TIP3 water molecules using the VMD software [55]. The dimensions of the system were 100 Å x 110 Å x 120 Å. All simulations were carried out using the parallel molecular dynamics program NAMD2 [41] and the CHARMM27 force field. Temperature was controlled by Langevin dynamics. Periodic boundary conditions were applied to obtain consistent behavior. The initial position of MTA on the extracellular side was then relaxed for 1 ns. The final state was saved as a restart point for the initial position of SMD. The pulling velocity was 10 Å/ns and a spring constant of 2.5 kcal/mol/Å^2^ was used. During each SMD, the force was only applied along the pulling direction. The trajectories were saved every 5 ps, and the steering forces were recorded every 0.5 ps. The trajectory along SERT was repeated four times. Finally, the force profile along the MTA pathway was constructed and 2000 MTA-SERT complex states (frames) were obtained from the SMD.

### Binding site alignment and common binding site generation

Structural alignments of the similar binding sites in MAO-A and SERT proteins were performed using the MultiBind computational method [56]. This approach reveals the common physicochemical patterns that may be responsible for the binding of the same ligand to different protein targets. For the recognition of common patterns, MultiBind carried out a multiple alignment between the binding sites defined by all residues of the MAO-A and SERT proteins that were located up to 4 Å away from MTA. Then, multiple structural rearrangements of superimposed binding sites were made using a Geometric Hashing technique [57]. Briefly and as previously described [51], this method consists of two main processes: a) the pre-processing of the features of each binding site conformation and hashing them into a table; and b) the recognition of the similar features in the objects of the hash table. In the pre-processing, each amino acid was denoted by pseudocenters (X, Y and Z coordinates) which provided a unique physicochemical property to the binding site: hydrogen-bond donor, hydrogen-bond acceptor, mixed donor/acceptor, hydrophobic aliphatic or aromatic contacts. Finally, MultiBind performed a combination of multiple superimposed binding site conformations in order to find consensus binding patterns. The pockets that originate the consensus binding site do not necessarily must have identical residues and therefore, different residues might be matched (aligned) if the overall structural alignment score between two pockets is better with that arrange. This procedure is similar to the Needleman-Wunsch pairwise alignment algorithm implemented in BLAST [58], but used in a 3D perspective. Then, the highest scored consensus binding conformations at the MAO-A and SERT proteins, which are stored as independent pdb files, were manually depurated. Thus, to generate a unique and common binding site, all equivalent amino acids (same physicochemical group: polar, non-polar, positively or negatively charged) that appeared superimposed in the binding sites found in each protein (both pdb files), were merged. In contrast, all non-equivalent amino acids were preserved in the final consensus binding site [51].

## Results and discussion

### Similarities between the dopamine and serotonin transporters

**Fig 3:**
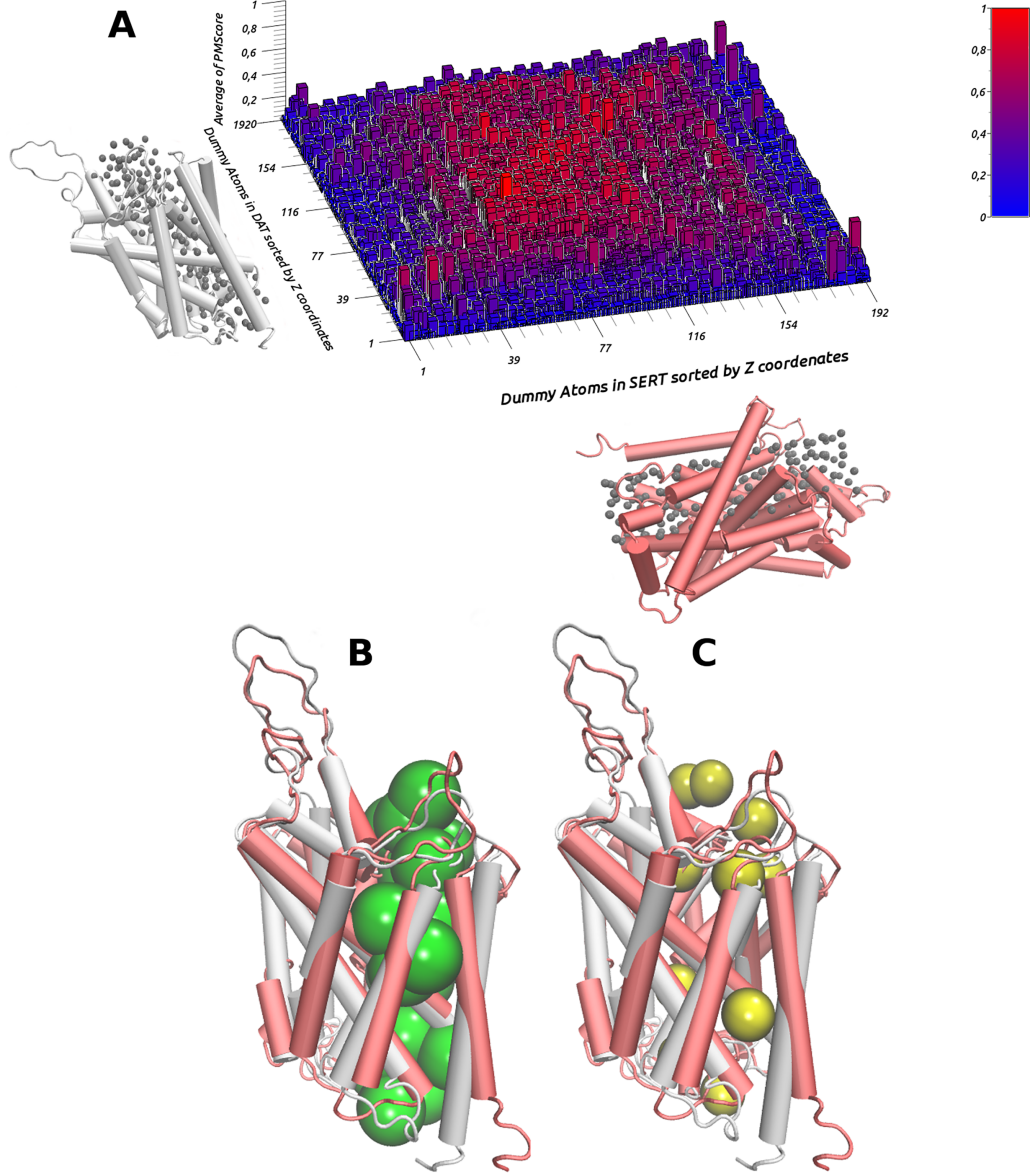
Average of similarity/differences between cavities in SERT and DAT. (A) shows the average of similarity between all patterns around each dummy atom in SERT (grey) versus all patterns around each dummy atom in DAT (red). The dummy atoms inserted in DAT are represented on the Y axis, sorted by the Z coordinate. The dummy atoms inserted in SERT are represented on the X axis, sorted by the Z coordinate. The average of the PMScore is represented on the Z axis. Each colored block represents the mean of the PMScores obtained after comparing all patterns (3–10 Å). Red represents high similarity scores (PMScore > 0.5). Blue represents low similarity scores (PMScore < 0.5). (B) and (C) show a superimposition of SERT and DAT and their detected similarities (green) and differences (yellow).

DAT and SERT share almost 70% of their amino acids sequences and their secondary and tertiary structures are relatively similar (RMSD = 3.0 Å; Figure G in S1 File) Furthermore, our partial 3D comparisons yielded, in many cases, PMScore values higher than 0.5, which are indicative of similarity. As shown in Fig 3A, a high degree of similarity (red blocks) appeared when comparing the cavities defined by the dummy atoms located in the transmembrane regions in both proteins. It is worth pointing out that the highest PMScore values (denoted in Fig 3A by the brightest red blocks) were obtained when the comparison was performed considering dummy atoms located at the same relative positions in both proteins (Fig 3B). On the contrary, the lowest PMScores values (PMScore ≤ 0.01; blue blocks Fig 3A) were observed when comparing the sites defined by the dummy atoms located at the intracellular and extracellular ends of the transporters, as illustrated in Fig 3C.

Overall, our results indicate that the SERT and the DAT have similar shapes and chemical features in their substrate permeation pathway, while structural differences occur in zones presumably associated with the initial recognition of selective ligands (extracellular and intracellular ends). Thus, these results yield insights regarding the structural features underlying the selectivity shown (either for uptake or reverse transport) by each protein for different substrates. Furthermore, the similarities found all along the pathway that is believed to be involved in the substrate transport process, agree with the idea that these proteins share a similar mechanism for the transport of substrates from the extracellular domain to the cytoplasm [5]. Interestingly, our findings might also account for the distinct transport rates that have been experimentally determined for diverse non-selective substrates [59].

### Similarities between MAO-B versus DAT and MAO-A versus SERT using dummy atoms

**Fig 4:**
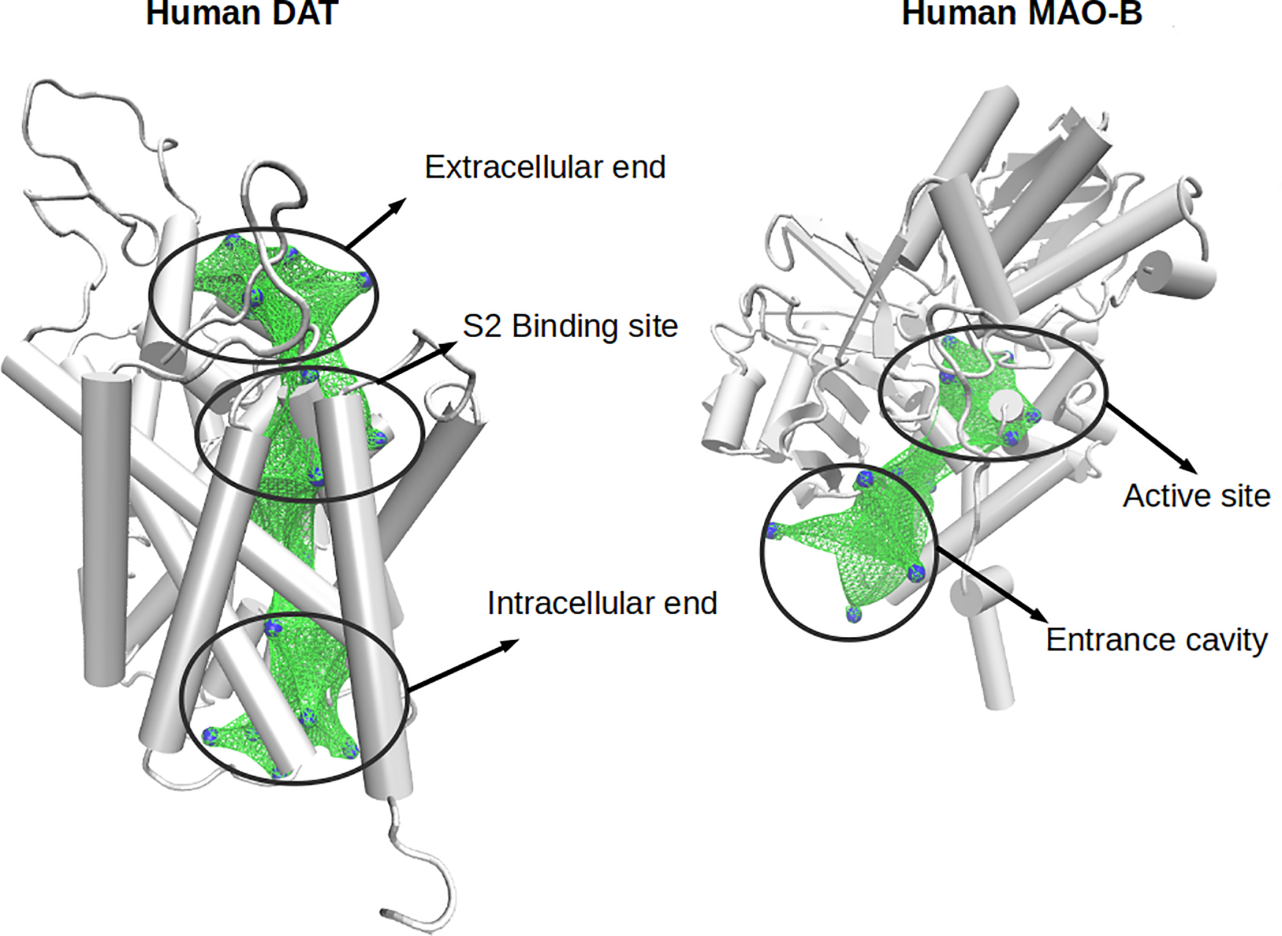
Similar zones between DAT and MAO-B. The 3D structures of DAT and MAO-B are shown in grey. Blue spheres represent the dummy atoms with an average PMScore greater than 0.5. Green surfaces are used to represent the similar zones.

**Fig 5:**
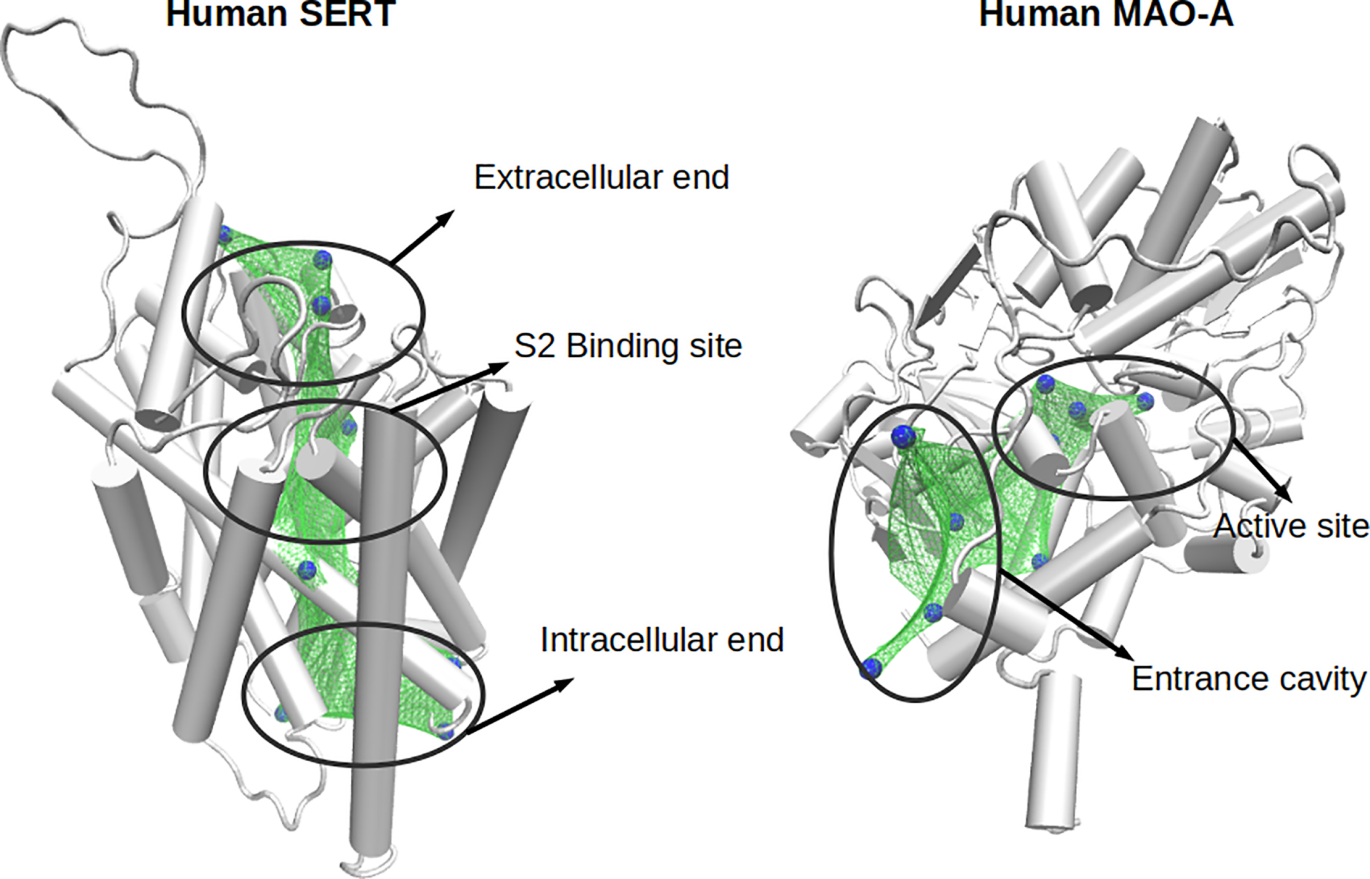
Similar zones between SERT and MAO-A. The 3D structures of SERT and MAO-A are shown in grey. Blue spheres represent the dummy atoms with an average PMScore greater than 0.5. Green surfaces represent the similar zones.

As illustrated in Figs 4 and 5, when comparing the cavities defined by dummy atoms in DAT and MAO-B, and those in SERT and MAO-A a few pairs of sites showed similarity values greater than 0.5 (denoted by bars in red; Figures H and I in S1 File). Interestingly, the pockets showing similarity appeared located at places where ligands must interact to be transported or metabolized by these proteins (e.g. the extracellular vestibule in DAT or SERT, and the entrance cavity or the catalytic site in MAO-B; Figs 4 and 5). Therefore, our results indicate that DAT and MAO-B, as well as SERT and MAO-A (whose preferential substrates are DA or 5-HT, respectively) exhibit striking structural similarities in zones presumably involved in the initial recognition of substrates. In addition, similarities were also found between the active (catalytic) site of MAOs and the extracellular vestibule in the transporters (also known as the S2 binding site; [60]). Indeed, additional comparisons between, SERT/MAO-B and DAT/MAO-A (as well as a reassessment of the similarities between SERT/MAO-A and DAT/MAO-B) were done using the ligand MTA docked into the S2 binding site of the transporters and into the active site of the MAOs. The results, obtained also with the PocketMatch algorithm, are shown in the Table A in S1 File. These data indicate that the binding sites of SERT/MAO-A and DAT/MAO-B are more similar than those in SERT/MAO-B or DAT/MAO-A, respectively. Interestingly, in these more local comparisons, all scores were higher than 0.5 (indicating more that 50% of structural similarity). This might be the reason why some ligands, *e*.*g*. amphetamine, are able to bind all of these target proteins. Thus, these results suggest the existence of a certain degree of structural convergence for proteins that have different functions, tissue distribution and genetic origin, but which share the same endogenous ligand. It is noteworthy that these similarities could not be identified using a sequence-based method, and although different algorithms and configurations were applied (local algorithms, global algorithms, low GAPs penalization, deleted GAP extension penalization, etc), no similarities were detected between MAOs and SERT/DAT (Figure J in S1 File). These results confirm the usefulness of the structure-based methods to find local similarities, where residues that form part of different binding sites are not located continuously in the primary sequences.

### Steered molecular dynamics (SMD) of the ligand 4-methylthioamphetamine (MTA) in SERT

**Fig 6:**
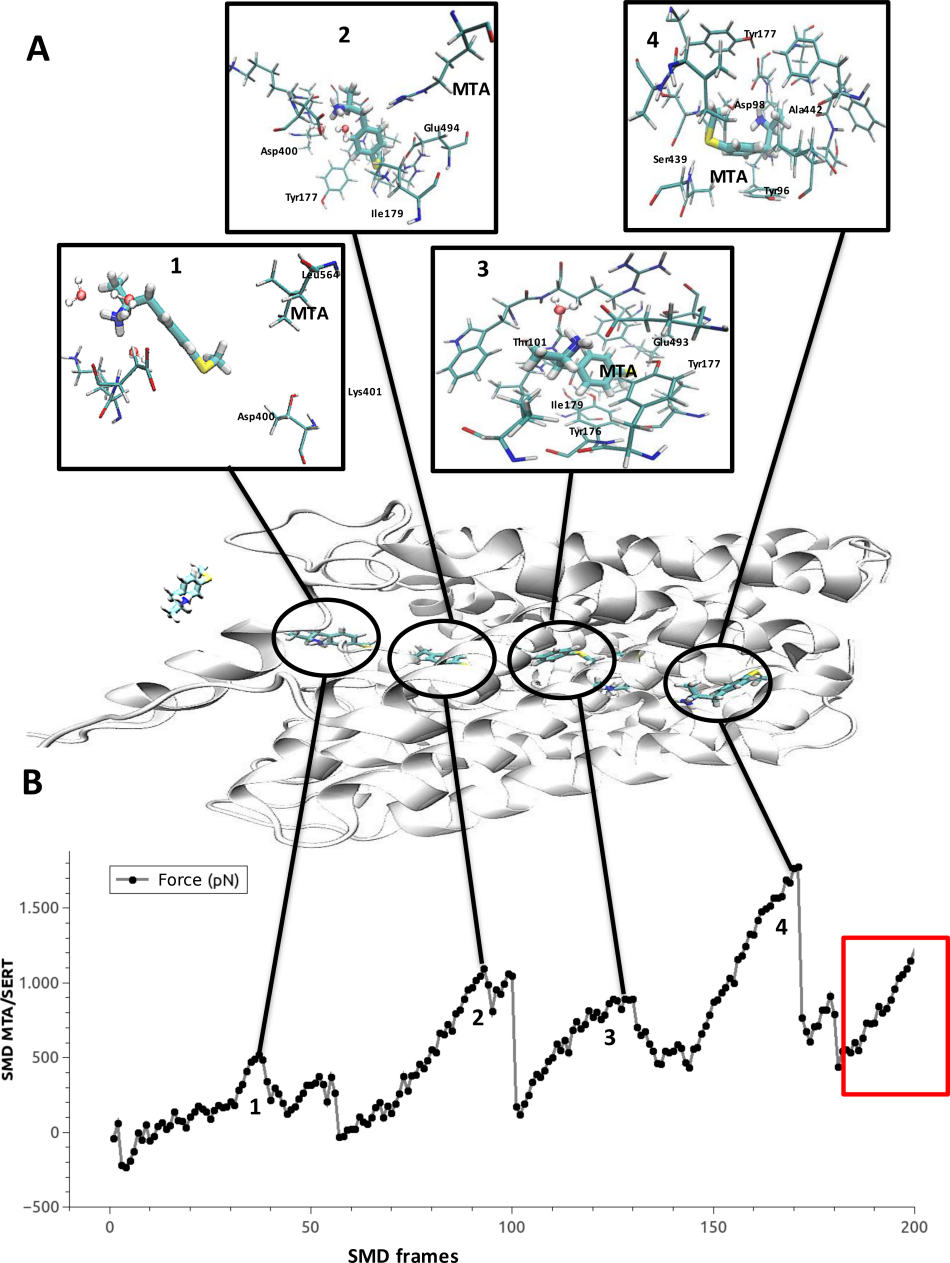
Steered molecular dynamics of MTA in SERT. Force profile (pN) versus the frame of the trajectory. Each force peak is represented with the localization of MTA and its interactions with SERT amino acid residues. The red square represents an artifact in the simulation.

Although our results showed significant similarities among binding sites present in all the proteins considered, these findings were obtained using dummy atoms. The use of these atoms does not consider conformational or electronic changes produced by ligands when interacting with their target structures. To address this issue, we simulated the presumable transport pathway of a substrate (in this case MTA) in SERT, and several possible binding sites (MTA-SERT complexes) were detected. As detailed in the methodology section, this was performed by forcing MTA to move from the extracellular to the intracellular domain of SERT, using an SMD simulation. Four distinct peaks of force were detected in the evaluation of the trajectory of MTA in SERT (Fig 6A, numbers 1–4). These force peaks indicate that more force was required to keep the velocity of MTA constant, and denote the formation of stable MTA-SERT complexes. The first peak occurred between frames 30 and 40 (number 1, Fig 6A and 6B) and represents the initial recognition site of MTA in the SERT. Here, a hydrogen-bond interaction between the amino group of MTA and Asp400 of SERT was identified. The second peak occurred between frames 90 and 100 (number 2, Fig 6A and 6B), which corresponds to the extracellular vestibule of SERT (the S2 binding site). Here, MTA is located in a position favorable to interact with residues such as Glu494, Tyr177 and Ile179. The third peak occurred between frames 120 and 130 (number 3, Fig 6A and 6B) and represents a putative MTA binding site that is located halfway between the S2 and the substrate binding site (also known as S1), but which still includes several residues of the extracellular vestibule of SERT. Here MTA showed a binding mode in which interactions could be established with Glu493, Ile179, Trp103, Tyr176 and Tyr177. These results are highly consistent with those recently reported for the binding and migration of 5-HT into SERT, as analyzed by computational methods [61].

It is noteworthy that in the analyses performed with the dummy atoms, the zone described by peaks 2 and 3 exhibited similarity with the substrate binding site of MAO-A. The fourth peak occurred between frames 150 and 180 (number 4, Fig 6A and 6B), and corresponds to the substrate binding site (also known as S1) of SERT. This site was also suggested as similar to the substrate binding site of MAO-A, according to our similarity determinations done using dummy atoms. Starting in frame 185, an abrupt change in the orientation of MTA was detected (red square Fig 6B). This behavior, which was not observed at any of the other peaks of force, was likely caused by a steric obstruction of SERT. This appears at the moment when a major conformational change (from an outward-facing to an inward-facing conformation) must occur in SERT in order to complete the proposed transport mechanism [62–64]. This major motion cannot be simulated by the SMD technique and more advanced approaches must be used to confirm this idea.

### Similarities between binding sites of MTA in MAO-A and several complexes of MTA-SERT from the SMD trajectory

**Fig 7:**
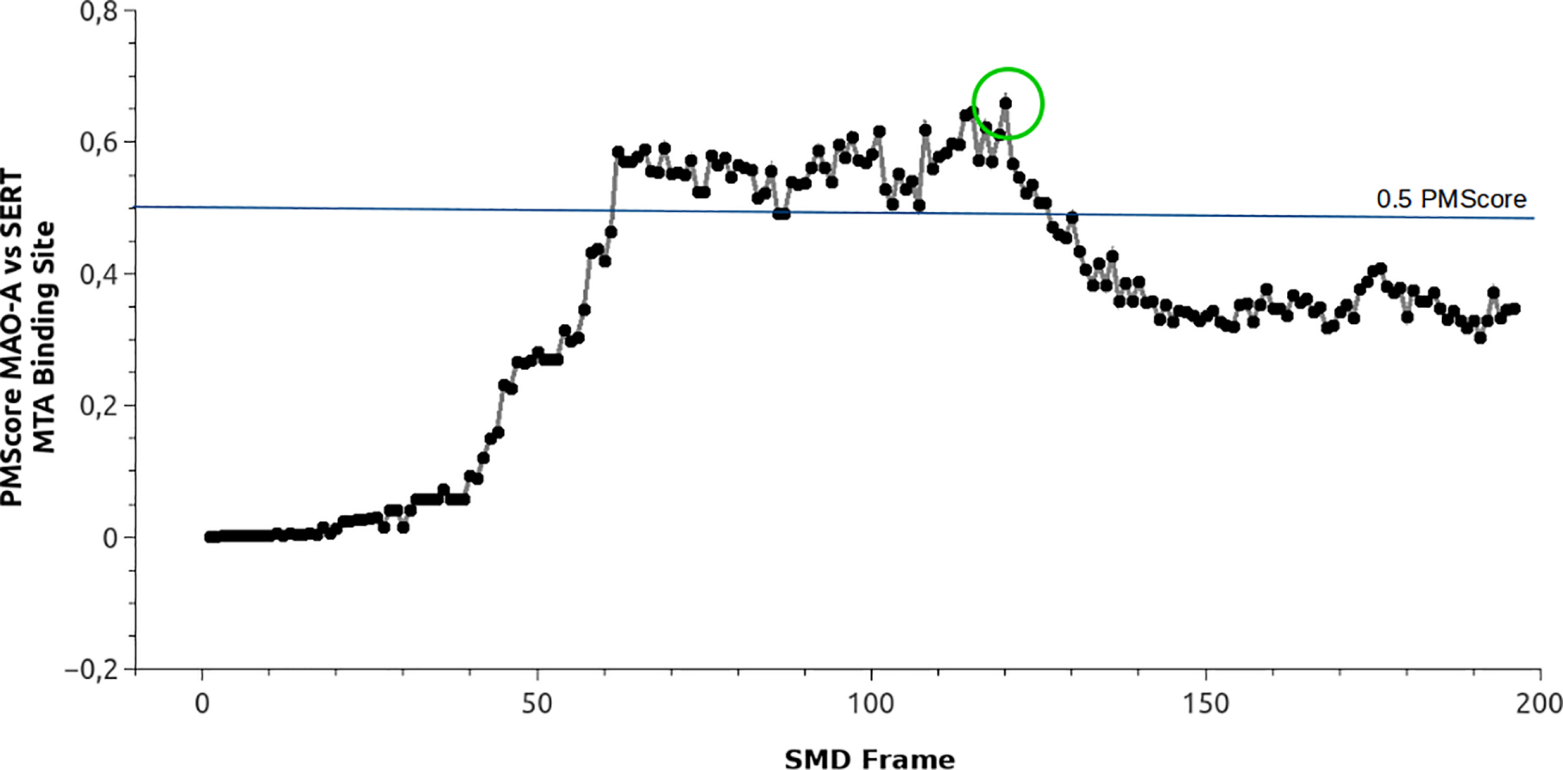
Profile of similarity between the binding site of MTA in MAO-A versus 200 putative binding sites of MTA in SERT. The Y axis represents the PMScore. The X axis represents an MTA/SERT complex from the SMD simulation.

Several putative binding sites of MTA were detected in SERT by the SMD simulations. Three of these (peaks 2, 3 and 4) concentrate the most relevant interactions and correspond to those reported as the binding sites (S2 and S1) of substrates and inhibitors in the monoamine transporters [10–12,18,65]. Additionally, these sites were classified as similar when compared with binding sites in the MAOs. As mentioned, the dummy atom method is a robust approximation but it does not consider the natural flexibility of proteins. This issue was improved with the inclusion of ligand based similarity measurements where 200 MTA-SERT complexes were compared with the MTA binding site in MAO-A, as determined by previous experimental and docking studies [54]. The results of this analysis are depicted in Fig 7. Interestingly, PMScores indicating similarity (i.e. > 0.5) were obtained when the binding sites detected in frames 70–130 (which include peaks 3 and 4 of Fig 6) were compared with the substrate binding site of MAO-A. Indeed, the highest PMScore value was detected in frame 120 (green circle in Fig 7). Considering that these results are in close agreement with those obtained when comparing MAO-A and SERT using dummy atoms, we propose that some shape and physicochemical features are conserved between the catalytic site of MAO-A and the extracellular vestibule of SERT. Moreover, these similarities might underlie the reasons for which a common ligand (e.g. 5-HT) is able to interact with both highly different proteins.

### Common binding site of MTA in MAO-A and SERT

**Fig 8:**
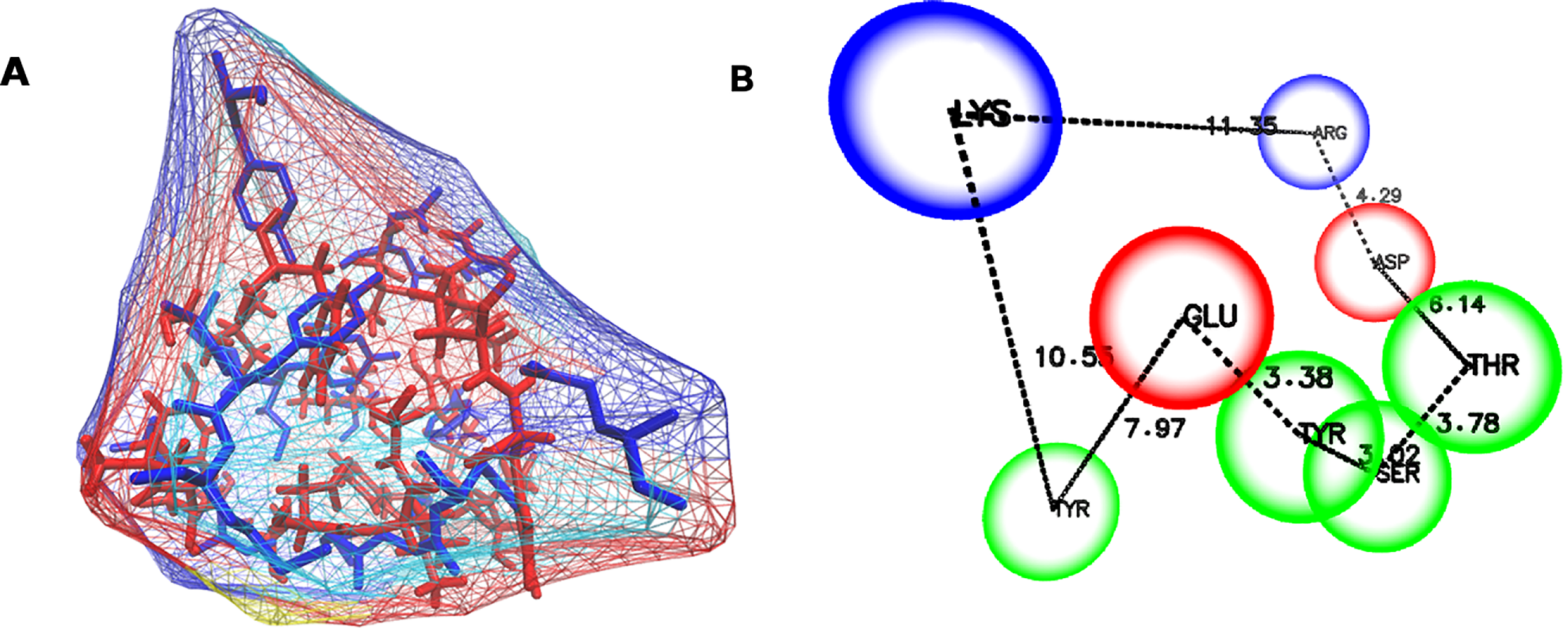
Common/consensus binding site of MTA in MAO-A and SERT. (A) shows the alignment of the binding sites and (B) shows the consensus binding site.

The binding site of MTA in MAO-A and the S2 binding site in SERT were structurally aligned using MultiBind software [56]. This approach revealed the common physicochemical patterns that might be responsible for the binding of the amphetamine derivative to both proteins. For the recognition of common patterns, MultiBind performed a multiple alignment between the binding sites defined by all residues of MAO-A and SERT located up to 6 Å from MTA. Several conformations were built and the most accurate alignment was identified (Fig 8A). Finally, and after a manual depuration, a unique common binding site was generated (Fig 8B).

The consensus binding site is formed by the following residues: Tyr, Glu, Asp, Gly, Arg, Thr and Ser (Fig 8B). It has a well-defined shape/cavity, and some chemical features such as two polar positively charged zones, two polar negatively charged zones and four aromatic non-polar components. These properties are in agreement with both the “aromatic cage” present in the catalytic site of MAOs [28] and the key residues Glu, Thr and Asp that form the S2 binding site of SERT. However, it should be noted that this consensus binding site must be cautiously considered in terms of drug design, since it might not reflect some specific physicochemical features of each isolated binding site. For instance, although negatively charged residues are contained in the common binding site, there are not such type of residues at the active site of MAO-A.

## Concluding remarks

3D similarities between SERT and DAT were found all along the pathway that is presumably involved in the substrate transport process. This agrees with the idea that these proteins share a similar mechanism for the transport of substrates from the extracellular domain to the cytoplasm [7,64]. In addition, 3D differences between SERT and DAT were found both at the extracellular and the intracellular ends of the transporters, in regions that, remarkably, are relatively distant from the S1 or S2 binding sites. These results are in agreement with recent computational and mutagenesis data showing that selective binding of substrates is not associated with the non-conserved SERT/DAT residues at S1 or S2 binding sites [66], but rather suggest that selectivity might be related to the initial recognition of substrates at the areas that we have found to show 3D differences. Altogether, similarities and differences detected when comparing SERT and DAT might be useful for both a better understanding of monoamine transporter function and for the design of selective and non-selective ligands.

Similarities were also found between the active (catalytic) site of MAO-A and the extracellular vestibule of SERT (the S2 binding site). These results suggest some degree of structural convergence [67] for these proteins which have different functions, tissue distribution and genetic origin, but which share the same endogenous ligand (5-HT). Hence, we propose the existence of a serotonergic “receptophore” in both proteins (the consensus binding site shown in Fig 8), which by analogy with the pharmacophore concept can be defined as a 3D ensemble, at the binding site(s) of two or more receptors, of molecular, steric and electronic features that ensure the optimal molecular interactions with a common promiscuous ligand. Further studies are necessary to determine if this 3D ensemble is also present in metabotropic and/or ionotropic 5-HT receptors, and if this concept can be extrapolated to other ligand-receptor systems.

Finally, from a methodological perspective, we want to emphasize that the use of dummy atoms instead of typical ligands to study binding site characteristics can be particularly appropriate in those cases where the ligand binding site is unknown.

## Supporting information

S1 File Supplementary information. Figure A. RMSD of MAO-A. Figure B. RMSD of MAO-B. Figure C. RMSD of SERT. Figure D: RMSD of DAT. Figure E: ProsaII evaluation of DAT. Figure F: Procheck evaluation of DAT. Figure G: RMSD between SERT and DAT. Figure H: Average of Similarity between all dummy atoms in DAT and MAO-B. Figure I: Average of Similarity between all dummy atoms in SERT and MAO-A. Table A: Binding sites similarities between MAOA/SERT, MAOA/DAT, MAOB/SERT and MAOB/DAT. Figure J: Representation of a sequence-based alignment between SERT and MAO-A.
(PDF)

## References

[1] Purves D, Augustine GJ, Fitzpatrick D, Hall WC, LaMantia A-S, McNamara JO WS, editor. Neuroscience. 3rd ed Sinauer Associates Sunderland; 2004.

[2] De Deurwaerdère P, Ramsay RR, Di Giovanni G. Neurobiology and neuropharmacology of monoaminergic systems. Prog Neurobiol. 2017 151: 1–3. 10.1016/j.pneurobio.2017.02.001 28259728

[3] Stahl SM. Stahl’s Essential Psychopharmacology. 4th ed Cambridge University Press; 2013.

[4] Kristensen AS, Andersen J, Jørgensen TN, Sørensen L, Eriksen J, Loland CJ, et al SLC6 Neurotransmitter Transporters: Structure, Function, and Regulation. Pharmacol Rev. 2011;63: 585–640. 10.1124/pr.108.000869 21752877

[5] Rudnick G, Krämer R, Blakely RD, Murphy DL, Verrey F. The SLC6 transporters: Perspectives on structure, functions, regulation, and models for transporter dysfunction. Pflugers Arch. 2014 pp. 25–42. 10.1007/s00424-013-1410-1 24337881 PMC3930102

[6] Bröer S, Gether U. The solute carrier 6 family of transporters. Br J Pharmacol. 2012 pp. 256–278. 10.1111/j.1476-5381.2012.01975.x 22519513 PMC3481037

[7] Penmatsa A, Gouaux E. How LeuT shapes our understanding of the mechanisms of sodium-coupled neurotransmitter transporters. J Physiol. 2014;592: 863–869. 10.1113/jphysiol.2013.259051 23878376 PMC3948551

[8] Loland CJ. The use of LeuT as a model in elucidating binding sites for substrates and inhibitors in neurotransmitter transporters. Biochimica et Biophysica Acta—General Subjects. 2015 pp. 500–510. 10.1016/j.bbagen.2014.04.011 24769398

[9] Yamashita A, Singh SK, Kawate T, Jin Y, Gouaux E. Crystal structure of a bacterial homologue of Na+/Cl—dependent neurotransmitter transporters. Nature. 2005;437: 215–23. 10.1038/nature03978 16041361

[10] Coleman JA, Green EM, Gouaux E. X-ray structures and mechanism of the human serotonin transporter. Nature. 2016;532: 334–339. 10.1038/nature17629 27049939 PMC4898786

[11] Wang KH, Penmatsa A, Gouaux E. Neurotransmitter and psychostimulant recognition by the dopamine transporter. Nature. 2015;521: 322–327. 10.1038/nature14431 25970245 PMC4469479

[12] Penmatsa A, Wang KH, Gouaux E. X-ray structures of Drosophila dopamine transporter in complex with nisoxetine and reboxetine. Nat Struct Mol Biol. 2015;22: 506–508. 10.1038/nsmb.3029 25961798 PMC4608549

[13] Grouleff J, Ladefoged LK, Koldsø H, Schiøtt B. Monoamine transporters: Insights from molecular dynamics simulations. Frontiers in Pharmacology. 2015 10.3389/fphar.2015.00235 26528185 PMC4607855

[14] Zhou Z, Zhen J, Karpowich NK, Goetz RM, Law CJ, Reith MEA, et al LeuT-desipramine structure reveals how antidepressants block neurotransmitter reuptake. Science (80-). 2007;317: 1390–1393. 10.1126/science.1147614 17690258 PMC3711652

[15] Singh SK, Yamashita A, Gouaux E. Antidepressant binding site in a bacterial homologue of neurotransmitter transporters. Nature. 2007;448: 952–956. 10.1038/nature06038 17687333

[16] Zhou Z, Zhen J, Karpowich NK, Law CJ, Reith MEA, Wang DN. Antidepressant specificity of serotonin transporter suggested by three LeuT-SSRI structures. Nat Struct Mol Biol. 2009;16: 652–657. 10.1038/nsmb.1602 19430461 PMC2758934

[17] Wang H, Goehring A, Wang KH, Penmatsa A, Ressler R, Gouaux E. Structural basis for action by diverse antidepressants on biogenic amine transporters. Nature. 2013;503: 141–145. 10.1038/nature12648 24121440 PMC3904662

[18] Penmatsa A, Wang KH, Gouaux E. X-ray structure of dopamine transporter elucidates antidepressant mechanism. Nature. 2013;503: 85–90. 10.1038/nature12533 24037379 PMC3904663

[19] Zhong H, Haddjeri N, Sánchez C. Escitalopram, an antidepressant with an allosteric effect at the serotonin transporter- -a review of current understanding of its mechanism of action. Psychopharmacology (Berl). 2012;219: 1–13. 10.1007/s00213-011-2463-5 21901317

[20] Plenge P, Shi L, Beuming T, Te J, Newman AH, Weinstein H, et al Steric hindrance mutagenesis in the conserved extracellular vestibule impedes allosteric binding of antidepressants to the serotonin transporter. J Biol Chem. 2012;287: 39316–39326. 10.1074/jbc.M112.371765 23007398 PMC3501018

[21] Edmondson DE, Binda C, Mattevi A. Structural insights into the mechanism of amine oxidation by monoamine oxidases A and B. Arch Biochem Biophys. 2007 pp. 269–276. 10.1016/j.abb.2007.05.006 17573034 PMC1993809

[22] Youdim MBH, Edmondson D, Tipton KF. The therapeutic potential of monoamine oxidase inhibitors. Nat Rev Neurosci. 2006;7: 295–309. 10.1038/nrn1883 16552415

[23] Binda C, Newton-Vinson P, Hubálek F, Edmondson DE, Mattevi A. Structure of human monoamine oxidase B, a drug target for the treatment of neurological disorders. Nat Struct Biol. 2002;9: 22–26. 10.1038/nsb732 11753429

[24] Binda C, Li M, Hubalek F, Restelli N, Edmondson DE, Mattevi A. Insights into the mode of inhibition of human mitochondrial monoamine oxidase B from high-resolution crystal structures. Proc Natl Acad Sci U S A. 2003;100: 9750–9755. 10.1073/pnas.1633804100 12913124 PMC187837

[25] Binda C, Hubálek F, Li M, Herzig Y, Sterling J, Edmondson DE, et al Crystal Structures of Monoamine Oxidase B in Complex with Four Inhibitors of the N-Propargylaminoindan Class. J Med Chem. 2004;47: 1767–1774. 10.1021/jm031087c 15027868

[26] Ma J, Yoshimura M, Yamashita E, Nakagawa A, Ito A, Tsukihara T. Structure of rat monoamine oxidase A and its specific recognitions for substrates and inhibitors. J Mol Biol. 2004;338: 103–114. 10.1016/j.jmb.2004.02.032 15050826

[27] Colibus L De, Li M, Binda C, Lustig A, Edmondson DE, Mattevi A. Three-dimensional structure of human monoamine oxidase A (MAO A): Relation to the structures of rat MAO A and human MAO B. 2005; 10.1073/pnas.0505975102 PMC1200291 16129825

[28] Binda C, Mattevi A, Edmondson DE. Structural properties of human monoamine oxidases A and B. Int Rev Neurobiol. 2011;100: 1–11. 10.1016/B978-0-12-386467-3.00001-7 21971000

[29] Reyes-Parada M., Iturriaga-Vasquez P., Fierro A, Cassels BK. Monoamine Oxidase Inhibition In the Light of New Structural Data. Curr Enzym Inhib. 2005;1: 85–95. 10.2174/1573408052952711

[30] Binda C, Newton-Vinson P, Hubálek F, Edmondson DE, Mattevi A. Structure of human monoamine oxidase B, a drug target for the treatment of neurological disorders. Nat Struct Biol. 2002;9: 22–26. 10.1038/nsb732 11753429

[31] De Colibus L, Li M, Binda C, Lustig A, Edmondson DE, Mattevi A. Three-dimensional structure of human monoamine oxidase A (MAO A): Relation to the structures of rat MAO A and human MAO B. Proc Natl Acad Sci U S A. 2005;102: 12684–12689. 10.1073/pnas.0505975102 16129825 PMC1200291

[32] Son S-Y, Ma J, Kondou Y, Yoshimura M, Yamashita E, Tsukihara T. Structure of human monoamine oxidase A at 2.2-A resolution: the control of opening the entry for substrates/inhibitors. Proc Natl Acad Sci U S A. 2008;105: 5739–5744. 10.1073/pnas.0710626105 18391214 PMC2311356

[33] Penmatsa A, Wang KH, Gouaux E. X-ray structure of dopamine transporter elucidates antidepressant mechanism. Nature. 2013; 1–12. 10.1038/nature12533 24037379 PMC3904663

[34] Wang H, Goehring A, Wang KH, Penmatsa A, Ressler R, Gouaux E. Structural basis for action by diverse antidepressants on biogenic amine transporters. Nature. 2013;503: 141–145. 10.1038/nature12648 24121440 PMC3904662

[35] Gani OA, Thakkar B, Narayanan D, Alam KA, Kyomuhendo P, Rothweiler U, et al Assessing protein kinase target similarity: Comparing sequence, structure, and cheminformatics approaches. Biochim Biophys Acta. 2015;1854: 1605–1616. 10.1016/j.bbapap.2015.05.004 26001898

[36] Vulpetti A, Kalliokoski T, Milletti F. Chemogenomics in drug discovery: computational methods based on the comparison of binding sites. Future Med Chem. 2012 pp. 1971–1979. 10.4155/fmc.12.147 23088277

[37] Jalencas X, Mestres J. Identification of Similar Binding Sites to Detect Distant Polypharmacology. Mol Inform. 2013;32: 976–990. 10.1002/minf.201300082 27481143

[38] Roche D, Brackenridge D, McGuffin L. Proteins and Their Interacting Partners: An Introduction to Protein–Ligand Binding Site Prediction Methods. Int J Mol Sci. 2015;16: 29829–29842. 10.3390/ijms161226202 26694353 PMC4691145

[39] Hidalgo S, Molina-Mateo D, Escobedo P, Zárate R V., Fritz E, Fierro A, et al Characterization of a Novel Drosophila SERT Mutant: Insights on the Contribution of the Serotonin Neural System to Behaviors. ACS Chem Neurosci. 2017; 10.1021/acschemneuro.7b00089 28665105

[40] Sotomayor-Zárate R, Quiroz G, Araya K a, Abarca J, Ibáñez MR, Montecinos A, et al 4-Methylthioamphetamine increases dopamine in the rat striatum and has rewarding effects in vivo. Basic Clin Pharmacol Toxicol. 2012;111: 371–9. 10.1111/j.1742-7843.2012.00926.x 22788961

[41] Nelson MT, Humphrey W, Gursoy A, Dalke A, Kale L V., Skeel RD, et al NAMD: a Parallel, Object-Oriented Molecular Dynamics Program. Int J High Perform Comput Appl. 1996 pp. 251–268. 10.1177/109434209601000401

[42] Wiederstein M, Sippl MJ. ProSA-web: Interactive web service for the recognition of errors in three-dimensional structures of proteins. Nucleic Acids Res. 2007;35 10.1093/nar/gkm290 17517781 PMC1933241

[43] Laskowski RA, MacArthur MW, Moss DS, Thornton JM. PROCHECK: a program to check the stereochemical quality of protein structures. J Appl Crystallogr. 1993 pp. 283–291. 10.1107/S0021889892009944

[44] Reyes-Parada M, Fierro a., Iturriaga-Vasquez P, Cassels B. Monoamine Oxidase Inhibition In the Light of New Structural Data. Curr Enzym Inhib. 2005;1: 85–95. 10.2174/1573408052952711

[45] Shi L, Quick M, Zhao Y, Weinstein H, Javitch JA. The mechanism of a neurotransmitter:sodium symporter—inward release of Na+ and substrate is triggered by substrate in a second binding site. Mol Cell. 2008;30: 667–677. 10.1016/j.molcel.2008.05.008 18570870 PMC2826427

[46] Penmatsa A, Wang KH, Gouaux E. X-ray structure of dopamine transporter elucidates antidepressant mechanism. Nature. 2013;503: 85–90. 10.1038/nature12533 24037379 PMC3904663

[47] McDonald GR, Olivieri A, Ramsay RR, Holt A. On the formation and nature of the imidazoline I2 binding site on human monoamine oxidase-B. Pharmacol Res. 2010;62: 475–488. 10.1016/j.phrs.2010.09.001 20832472

[48] Bonivento D, Milczek EM, McDonald GR, Binda C, Holt A, Edmondson DE, et al Potentiation of ligand binding through cooperative effects in monoamine oxidase B. J Biol Chem. 2010;285: 36849–36856. 10.1074/jbc.M110.169482 20855894 PMC2978614

[49] Yeturu K, Chandra N. PocketMatch: a new algorithm to compare binding sites in protein structures. BMC Bioinformatics. 2008;9: 543 10.1186/1471-2105-9-543 19091072 PMC2639437

[50] Fierro A, Montecinos A, Gomez-Molina C, Nunez G, Aldeco M, Edmondson Dale E, et al Similarities Between the Binding Sites of Monoamine Oxidase (MAO) from Different Species—Is Zebrafish a Useful Model for the Discovery of Novel MAO Inhibitors?, An Integrated View of the Molecular Recognition and Toxinology—From Analytical Procedures. Intech; 2013 pp. 405–431.

[51] Möller-Acuña P, Contreras-Riquelme JS, Rojas-Fuentes C, Nuñez-Vivanco G, Alzate-Morales J, Iturriaga-Vásquez P, et al Similarities between the binding sites of SB-206553 at serotonin type 2 and alpha7 acetylcholine nicotinic receptors: Rationale for its polypharmacological profile. PLoS One. 2015;10 10.1371/journal.pone.0134444 26244344 PMC4526571

[52] Gobbi M, Moia M, Pirona L, Ceglia I, Reyes-Parada M, Scorza C, et al p-methylthioamphetamine and 1-(m-chlorophenyl)piperazine, two non-neurotoxic 5-HT releasers in vivo, differ from neurotoxic amphetamine derivatives in their mode of action at 5-HT nerve endings in vitro. J Neurochem. 2002;82: 1435–1443. 10.1046/j.1471-4159.2002.01073.x 12354291

[53] Scorza MC, Carrau C, Silveira R, Zapata-Torres G, Cassels BK, Reyes-Parada M. Monoamine oxidase inhibitory properties of some methoxylated and alkylthio amphetamine derivatives. Structure-activity relationships. Biochem Pharmacol. 1997;54: 1361–1369. 10.1016/S0006-2952(97)00405-X 9393679

[54] Fierro A, Osorio-Olivares M, Cassels BK, Edmondson DE, Sepúlveda-Boza S, Reyes-Parada M. Human and rat monoamine oxidase-A are differentially inhibited by (S)-4-alkylthioamphetamine derivatives: Insights from molecular modeling studies. Bioorganic Med Chem. 2007;15: 5198–5206. 10.1016/j.bmc.2007.05.021 17521909 PMC1949415

[55] Humphrey W, Dalke A, Schulten K. VMD: Visual molecular dynamics. J Mol Graph. 1996;14: 33–38. 10.1016/0263-7855(96)00018-5 8744570

[56] Shulman-Peleg A, Shatsky M, Nussinov R, Wolfson HJ. MultiBind and MAPPIS: webservers for multiple alignment of protein 3D-binding sites and their interactions. Nucleic Acids Res. 2008;36 10.1093/nar/gkn185 18467424 PMC2447750

[57] Maurer WD, Lewis TG. Hash Table Methods. ACM Comput Surv. 1975;7: 5–19. 10.1145/356643.356645

[58] Altschul SF, Gish W, Miller W, Myers EW, Lipman DJ. Basic local alignment search tool. Journal of Molecular Biology. 1990 pp. 403–410. 10.1016/S0022-2836(05)80360-2 2231712

[59] Larsen MB, Sonders MS, Mortensen OV, Larson GA, Zahniser NR, Amara SG. Dopamine transport by the serotonin transporter: a mechanistically distinct mode of substrate translocation. J Neurosci. 2011;31: 6605–6615. 10.1523/JNEUROSCI.0576-11.2011 21525301 PMC3107525

[60] Haddad Y, Heger Z, Adam V. Guidelines for Homology Modeling of Dopamine, Norepinephrine, and Serotonin Transporters. ACS Chem Neurosci. 2016;7: 1607–1613. 10.1021/acschemneuro.6b00242 27596073

[61] Topiol S, Bang-Andersen B, Sanchez C, Bøgesø KP. Exploration of insights, opportunities and caveats provided by the X-ray structures of hSERT. Bioorganic Med Chem Lett. 2016;26: 5058–5064. 10.1016/j.bmcl.2016.08.087 27624075

[62] Rudnick G. Cytoplasmic permeation pathway of neurotransmitter transporters. Biochemistry. 2011 pp. 7462–7475. 10.1021/bi200926b 21774491 PMC3164596

[63] Krishnamurthy H, Gouaux E. X-ray structures of LeuT in substrate-free outward-open and apo inward-open states. Nature. Nature Publishing Group; 2012;481: 469–74. 10.1038/nature10737 22230955 PMC3306218

[64] Kazmier K, Claxton DP, Mchaourab HS. Alternating access mechanisms of LeuT-fold transporters: trailblazing towards the promised energy landscapes. Curr Opin Struct Biol. 2017 pp. 100–108. 10.1016/j.sbi.2016.12.006 28040635 PMC5491374

[65] Wang H, Goehring A, Wang KH, Penmatsa A, Ressler R, Gouaux E. Structural basis for action by diverse antidepressants on biogenic amine transporters. Nature. 2013;503: 141–5. 10.1038/nature12648 24121440 PMC3904662

[66] Andersen J, Ladefoged LK, Kristensen TNB, Munro L, Grouleff J, Stuhr-Hansen N, et al Interrogating the Molecular Basis for Substrate Recognition in Serotonin and Dopamine Transporters with High-Affinity Substrate-Based Bivalent Ligands. ACS Chem Neurosci. 2016;7: 1406–1417. 10.1021/acschemneuro.6b00164 27425420

[67] Najmanovich RJ. Evolutionary studies of ligand binding sites in proteins. Curr Opin Struct Biol. 2017 pp. 85–90. 10.1016/j.sbi.2016.11.024 27992825

