# Supplementary material for: 3D similarities between the binding sites of monoaminergic target proteins
Source: PLoS One. 2018 Jul 20;13(7):e0200637. doi: 10.1371/journal.pone.0200637 (PMC6054423; doi:10.1371/journal.pone.0200637)
Supplement: S1 File — Figure A. RMSD of MAO-A. Figure B. RMSD of MAO-B. Figure C. RMSD of SERT. Figure D: RMSD of DAT. Figure E: ProsaII evaluation of DAT. Figure F: Procheck evaluation of DAT. Figure G: RMSD between SERT and DAT. Figure H: Average of Similarity between all dummy atoms in DAT and MAO-B. Figure I: Average of Similarity between all dummy atoms in SERT and MAO-A. Table A: Binding sites similarities between MAOA/SERT, MAOA/DAT, MAOB/SERT and MAOB/DAT. Figure J: Representation of a sequence-based alignment between SERT and MAO-A. (PDF) [file pone.0200637.s001.pdf]

Supplementary Information

Figure A: RMSD of MAO-A

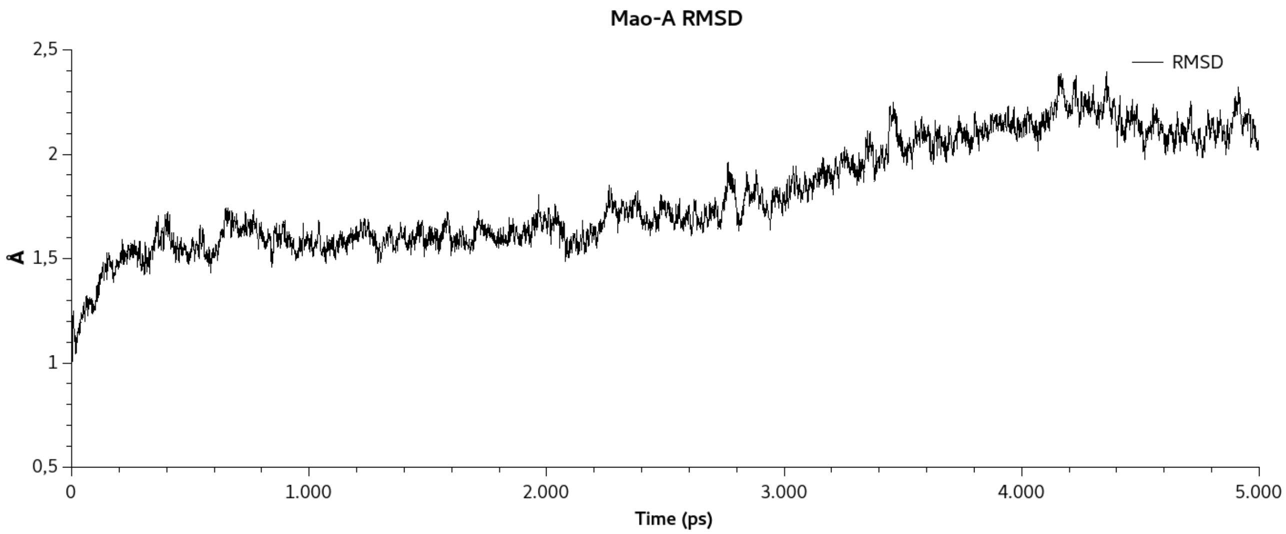

Figure A shows the RMSD of MAO-A in a simulation of 5 ns

Figure B: RMSD of MAO-B

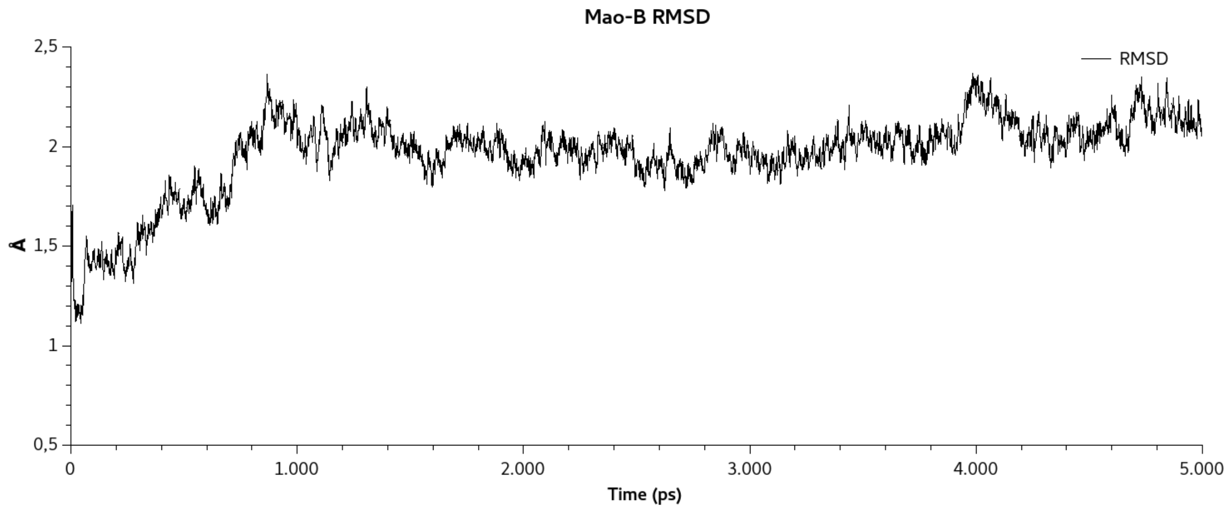

Figure B shows the RMSD of MAO-B in a simulation of 5 ns

Figure C: RMSD of SERT

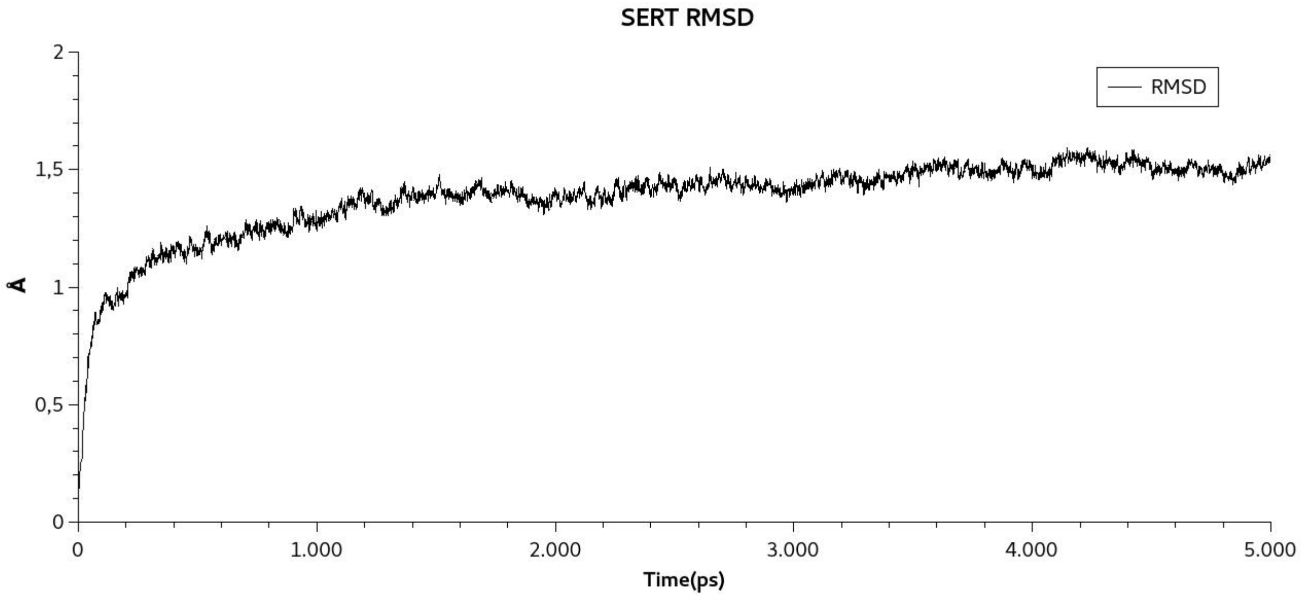

Figure C shows the RMSD of SERT in a simulation of 5 ns

Figure D: RMSD of DAT

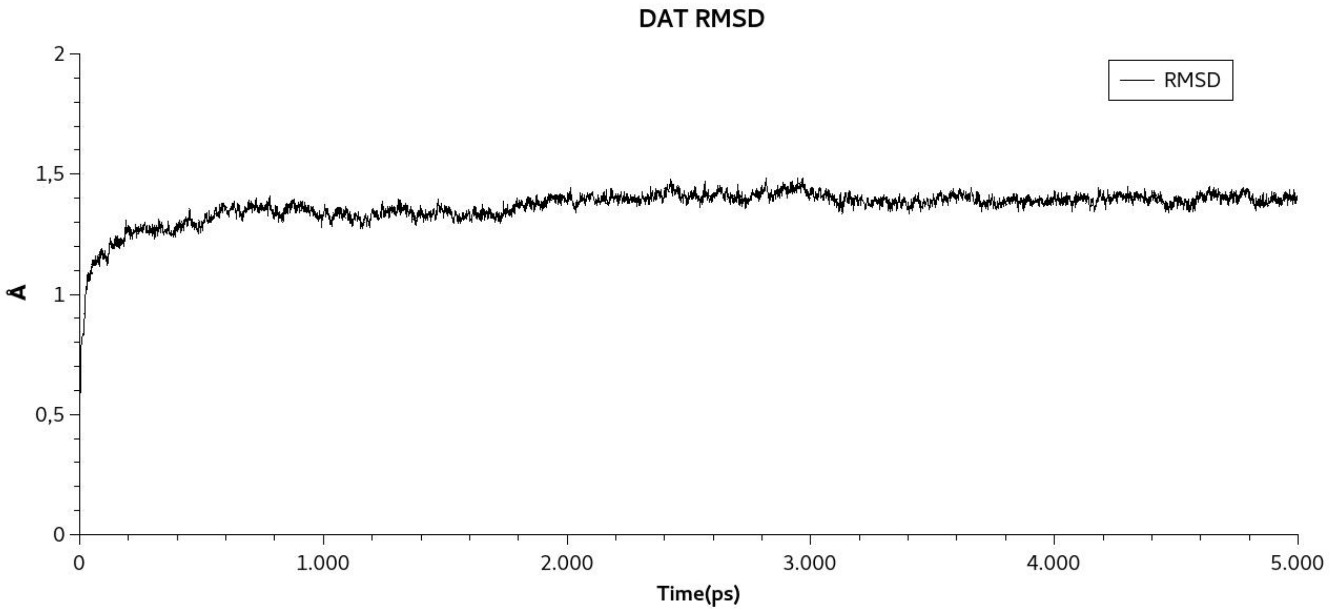

Figure D shows the RMSD of DAT in a simulation of 5 ns

Figure E: ProsaII evaluation of DAT

A

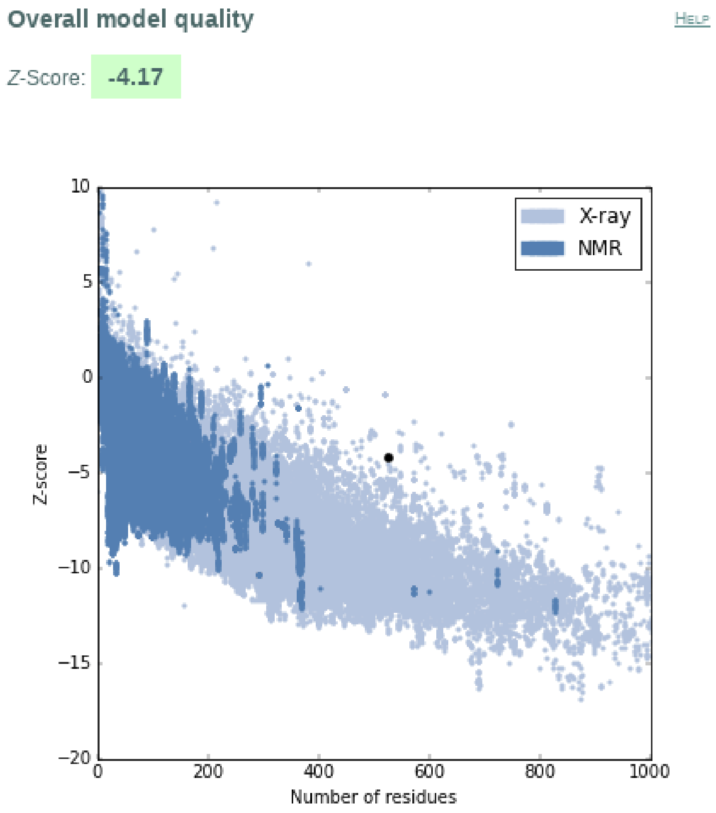

B

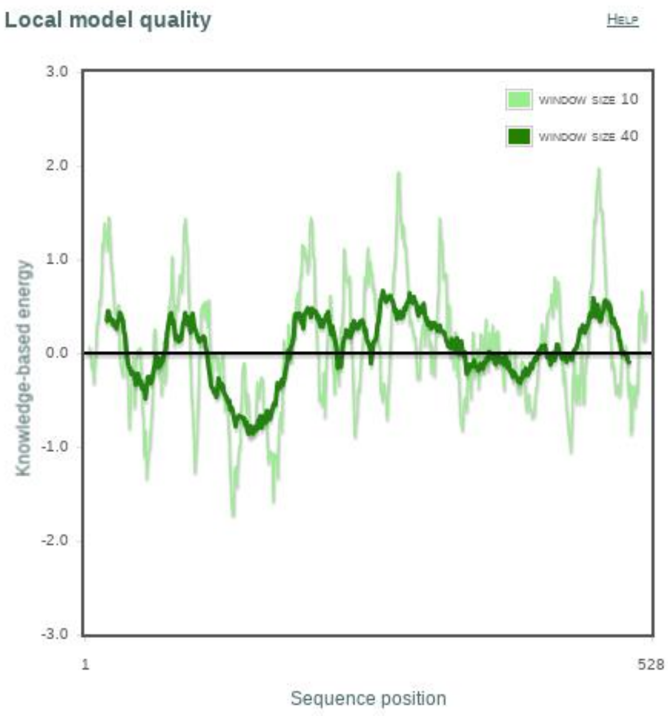

Figure E shows the evaluation of the homology model of the human DAT

Figure F: Procheck evaluation of DAT

1. Ramachandran Plot statistics

		No. of residues	%-tage
Most favoured regions	[A,B,L]	422	92.5%
Additional allowed regions	[a,b,l,p]	33	7.2%
Generously allowed regions	[~a,~b,~l,~p]	1	0.2%
Disallowed regions	[XX]	0	0.0%
Non-glycine and non-proline residues		456	100.0%
End-residues (excl. Gly and Pro)		2	
Glycine residues		47	
Proline residues		23	
Total number of residues		528	

2. G-Factors

Parameter	Score	Average Score
Dihedral angles:-		
Phi-psi distribution	0.08	
Chi1-chi2 distribution	-0.43	
Chi1 only	-0.15	
Chi3 & chi4	0.56	
Omega	**-1.07****	
		-0.39
Main-chain covalent forces:-		
Main-chain bond lengths	**-1.27****	
Main-chain bond angles	**-1.37****	
		**-1.32****
OVERALL AVERAGE		-0.24

Figure F shows the evaluation of the homology model of the human DAT

Figure G: RMSD between SERT and DAT

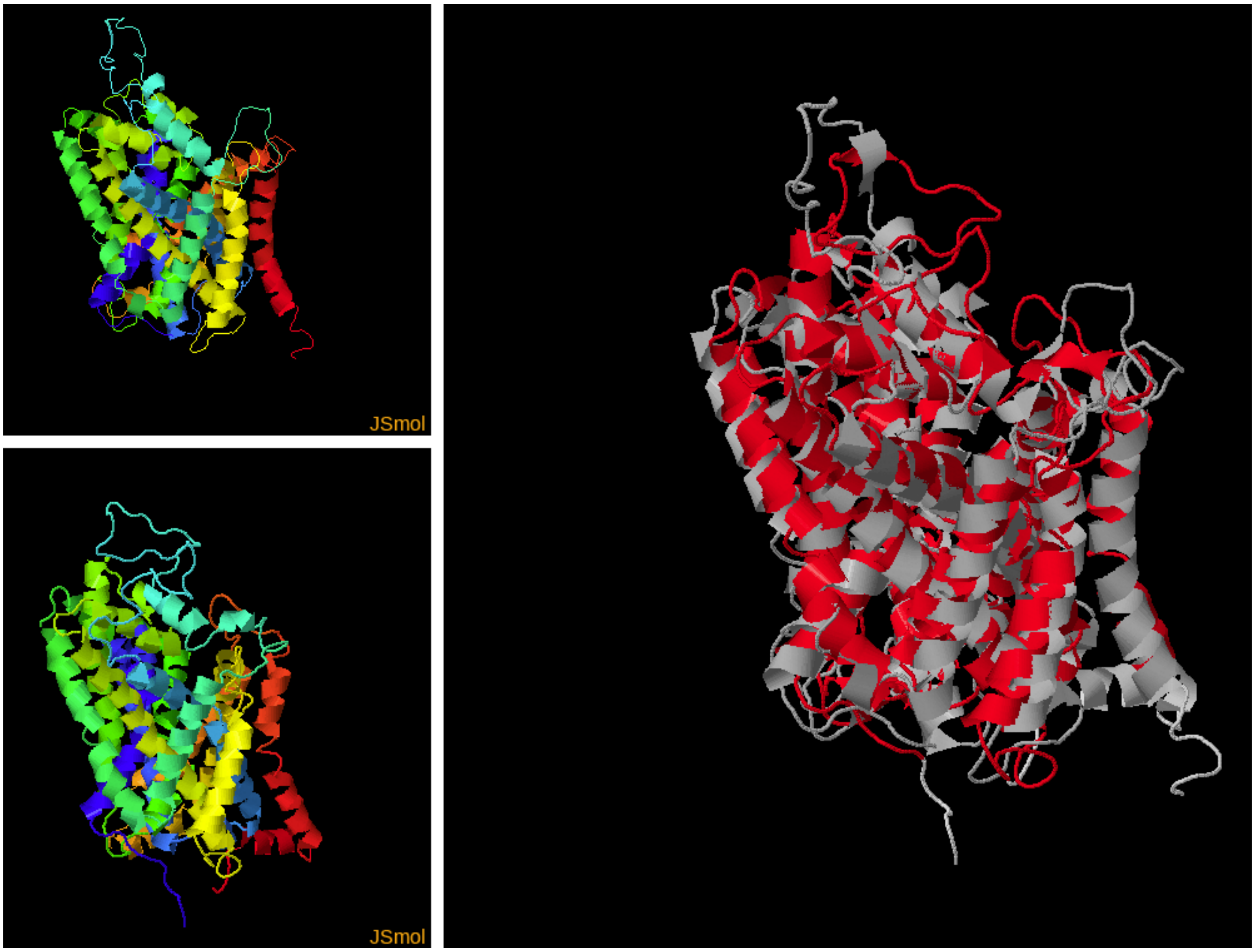

Figure G shows the structure alignment of SERT and DAT, which contains 466 equivalent positions with an RMSD of 3.0 Å (left top: DAT, left bottom: SERT). The structure alignment and the RMSD value were obtained using FatCat server (http://fatcat.burnham.org)

Figure H: Average of Similarity between all dummy atoms in DAT and MAO-B

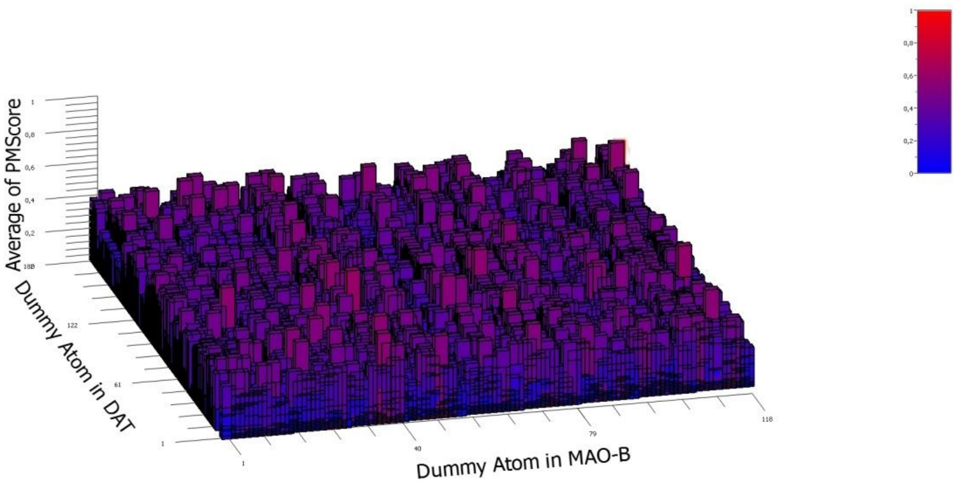

Figure H shows the average of similarity between all patterns around each dummy atom in DAT versus all patterns around each dummy atom in MAO-B (red). The dummy atoms inserted in DAT are represented on the Y axis, sorted by the Z coordinate. The dummy atoms inserted in MAO-B are represented on the X axis, sorted by the Z coordinate. The average of the PMScore is represented on the Z axis. Each colored block represents the mean of the PMScores obtained after comparing all patterns (3-10 Å). Red represents high similarity scores (PMScore > 0.5). Blue represents low similarity scores (PMScore < 0.5).

Figure I: Average of Similarity between all dummy atoms in SERT and MAO-A.

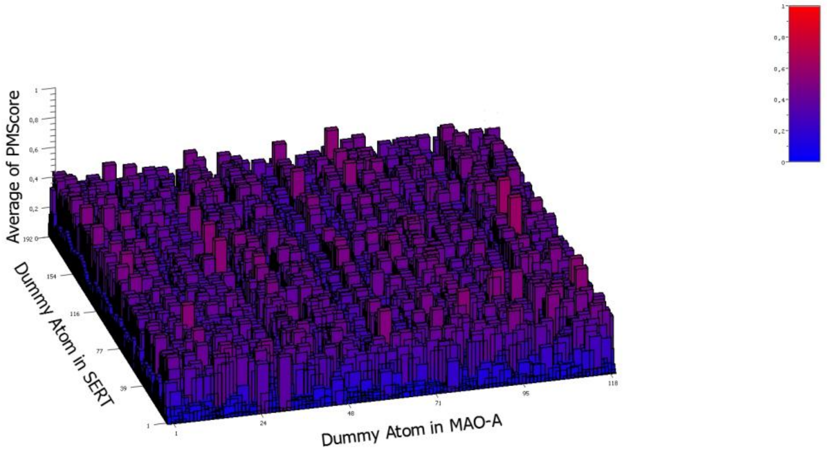

Figure I shows the average of similarity between all patterns around each dummy atom in SERT versus all patterns around each dummy atom in MAO-A (red). The dummy atoms inserted in SERT are represented on the Y axis, sorted by the Z coordinate. The dummy atoms inserted in MAO-A are represented on the X axis, sorted by the Z coordinate. The average of the PMScore is represented on the Z axis. Each colored block represents the mean of the PMScores obtained after comparing all patterns (3-10 Å). Red represents high similarity scores (PMScore > 0.5). Blue represents low similarity scores (PMScore < 0.5).

Table A: Binding sites similarities between MAOA/SERT, MAOA/DAT, MAOB/SERT and MAOB/DAT

Protein A	Protein B	PMScore
Mao-A	SERT	78,05 %
Mao-B	SERT	68,94 %
Mao-A	DAT	92,61 %
Mao-B	DAT	95,92 %

Table A shows the PMScore using MTA as ligand between the active site of MAOs and the S2 binding site of the transporters.

Figure J: Representation of a sequence-based alignment between SERT and MAO-A.

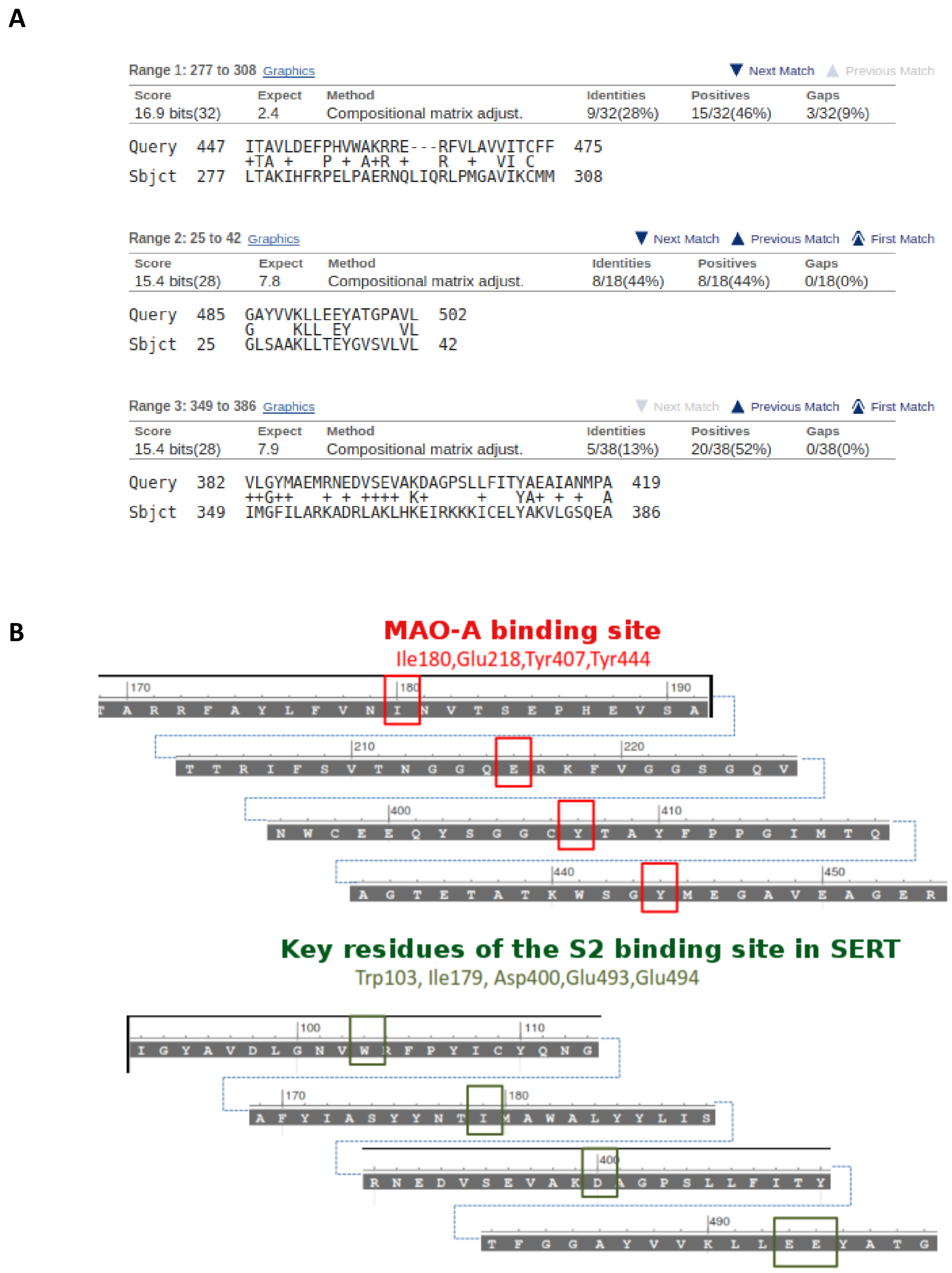

A) Blastp alignment between human SERT and MAO-A. B) Some key residues of the binding sites of MAO-A and SERT are remarked in boxes.
